# Costs and Resource Use Among Patients with Cervical Cancer, Cervical Intraepithelial Neoplasia, and Genital Warts in Algeria

**DOI:** 10.36469/jheor.2022.31049

**Published:** 2022-02-07

**Authors:** Ali-Chakib Bennacef, Aomar Ammar Khodja, Fadl Allah Abou-Bekr, Tidiane Ndao, Ryan Holl, Goran Benčina

**Affiliations:** 1 Merck Sharp & Dohme, Algeria; 2 University Hospital Mustapha Bacha, Algiers, Algeria; 3 University Hospital of Sidi Bel Abbes, Algeria; 4 Merck Sharp & Dohme, Morocco; 5 Merck Sharp & Dohme International GmbH, Kriens, Switzerland; 6 Center for Observational and Real-world Evidence, Merck Sharp & Dohme, Madrid, Spain

**Keywords:** papillomavirus infections, sexually transmitted viral diseases, genital warts, North Africa epidemiology, Algeria epidemiology, burden of illness, cost of illness

## Abstract

**Background:** Cervical cancer rates in North Africa have risen in the last 10 years, suggesting that this region might benefit from cervical cancer screening and HPV vaccination programs. To assess the potential benefits of cervical cancer screening and HPV vaccination in North African countries, country-specific data on the prevalence and burden of HPV-related conditions are needed.

**Objectives:** To describe the patterns and estimate the costs of management of cervical cancer, cervical intraepithelial neoplasia (CIN), and genital warts in Algeria.

**Methods:** This was a descriptive analysis of questionnaire data obtained from a panel of 15 oncologists, gynecologists, and dermatologists (n=5 each). Data on diagnostic and treatment patterns, recurrence, and healthcare resource use (HCRU) were obtained. The costs (in Algerian dinars) associated with diagnosis, treatment, and recurrence were estimated.

**Results:** Diagnosis of CIN was obtained by cytology tests or lesion biopsies; for cervical cancer, lesion biopsies, MRI, and CT scans were the most common diagnostic tests. For CIN, 70% of gynecologists and/or oncologists regularly or always used conization as a treatment. Treatments used regularly or always for cervical cancer included chemotherapy (80%), hysterectomy (70%), and radiation (70%). Annual HCRU per institution included 20 outpatient visits and 15 hospitalizations for CIN, and 50 outpatient visits and 11 hospitalizations for cervical cancer. For genital warts, diagnostic tests performed regularly or always included assays for hepatitis B, hepatitis C, HIV, and syphilis; cervical cytology; and colposcopy. Cryotherapy was the universal first-line treatment. Median per-patient costs associated with diagnosis, treatment, and recurrence were 6750, 19 750, and 77 750, respectively, for CIN; 53 750, 650 000, and 431 250, respectively, for cervical cancer; and 16 075, 15 500, and 9250, respectively, for genital warts.

**Discussion:** These results give an estimate of the HCRU and cost of cervical cancer, CIN, and genital warts and highlight the need to assess more precisely the epidemiology of these diseases in Algeria.

**Conclusions:** This study investigated the management of patients with cervical cancer, CIN, or genital warts in Algeria and provided the first estimates of diagnosis and treatment patterns, HCRU, and costs associated with these conditions. These resource use and cost estimates highlight the need to develop prevention strategies for HPV-related pathologies.

## INTRODUCTION

Human papillomavirus (HPV) is a sexually transmitted virus that infects >10% of women and ≥19% of men globally.^1^ High-risk HPV genotypes 16 and 18 are the cause of approximately 70% of cervical cancers, and low-risk genotypes 6 and 11 are associated with 90% of genital warts cases.^2^ Programs for cervical cancer screening and vaccination against HPV have been shown to reduce population levels of cervical cancer and genital warts.^3–5^

Relative to the rest of the world, North Africa is a low-risk region regarding cervical cancer,^6,7^ and HPV prevalence is also low.^1,8^ However, cervical cancer rates in North Africa have risen in the last 10 years (from 6.6 to 7.2 cases per 100 000),^6,9^ suggesting that this region might benefit from cervical cancer screening and HPV vaccination programs. Indeed, a cost-effectiveness analysis of the extended Middle East and North Africa predicted that ~180 000 cases of cervical cancer would be averted over the lifetime of 5 consecutive birth cohorts upon implementation of cervical cancer screening and HPV vaccination.^10^ For Algeria, where the age-standardized cervical cancer incidence is 8.1 per 100 000,^11^ this model projected that the risk of cervical cancer would be reduced 46.7% by conducting cervical cancer screening every 3 years, and by 75.3% with cervical cancer screening plus HPV vaccination.^10^

To assess the potential benefits of cervical cancer screening and HPV vaccination in North African countries, country-specific data on the prevalence and burden of HPV-related conditions are needed. Currently, there are no data on the healthcare resource utilization (HCRU) patterns among patients diagnosed with cervical cancer, cervical neoplasia, or genital warts in Algeria. Similarly, the economic impact of these diseases on patients and the healthcare system in Algeria is unknown. The purpose of this study was to describe the patterns and estimate the costs of management of cervical cancer, cervical intraepithelial neoplasia (CIN), and genital warts in Algeria.

## METHODS

### Study Design

This was a descriptive analysis of questionnaire data obtained via a Delphi panel. A Delphi panel was chosen because there is a lack of reliable chart data for the management of genital warts in Algeria. In addition, electronic medical reports are not yet available in the country. Questionnaires were completed by participating physicians from December 2019 to March 2020 in 2 rounds. The study protocol was approved by the Beni Messous Ethics Committee on December 12, 2019.

### Study Sample

There is no optimal number of participants on a Delphi panel in the literature; for feasibility, we chose to recruit 15 experts in the fields of cervical cancer, CIN, and genital warts in Algeria, specifically, oncologists, gynecologists, and dermatologists. Individual subject matter experts were identified via publication records, lists of conference organizers and presenters, and membership in relevant medical associations. Qualifying physicians had to (1) be based in Algeria; (2) have an active practice where patients with cervical cancer, CIN, and genital warts were treated; and (3) work at least part-time in the public sector. Individuals who (1) might be involved in the inclusion of HPV vaccination in the national immunization program, (2) only worked in private medical offices and/or private clinics, or (3) did not complete the confidentiality disclosure agreement for the study were excluded.

### Data Collection

After identifying a list of 15 potential subject matter experts among department heads at the 14 academic hospitals in Algeria, the third-party vendor contacted each one, requesting their participation in the Delphi panel. Panelists were informed prior to their engagement that Merck Sharp & Dohme, Algeria was sponsoring the research. All 15 experts agreed to participate, and an initiation visit was performed for each site to describe the Delphi technique and the reimbursement offered for participation (25.35 Algerian dinars [DZD]). The initiation visit concluded with a request to complete the initial study questionnaire (on paper). The second round of the Delphi process was done via email and consisted of circulation of the results of round 1, along with a request for panelists to review and confirm their answers from round 1. Panelists were responsible for recording and verifying the accuracy of their data. Data from the paper copies of the questionnaires were entered into a secure electronic database by the third-party vendor.

### Questionnaire Content

The questionnaires are shown in the **Appendix**. Questions about CIN/cervical cancer were directed to gynecologists/oncologists; questions about genital warts were directed to dermatologists. Main themes of each questionnaire were (1) panelists’ demographic characteristics (eg, sex, age, medical specialty, years of experience); (2) characteristics of panelists’ practices pertaining to either CIN/cervical cancer (for gynecologists/oncologists) or genital warts (for dermatologists) (eg, number of patients seen per month, distribution of patients in terms of sex, age, stage [for CIN], and duration of care); (3) institutional information (eg, availability of treatment guidelines, commonly used diagnostic tests, and commonly used treatments); and (4) HCRU (eg, hospitalizations, including length of stay, and outpatient visits). The questionnaire also asked about side effects and complications of treatment, referrals to other medical specialties, frequency and duration of follow-up, and recurrences. Questions generally took one of two forms, asking either for a number (eg, percentage of patients, duration of treatment in years, estimate of prevalence, etc) or a categorical designation (eg, yes/no; or never, sometimes, regularly, or always).

### Statistical Analysis

Answers to the questionnaire were analyzed descriptively using SAS 9.4/STAT 15.3 (SAS Institute, NC, USA). Continuous quantitative variables were described by the number of documented values, number of missing values, mean, standard deviation of the mean, median, first and third quartiles (interquartile range [IQR]), and range. Due to the small sample size, the results for continuous variables are generally reported in this paper as the median and IQR, with exceptions indicated in the table footnotes. Categorical variables were described by the number and the percentage of answers in each category, with the latter presented in the text.

### Cost Estimation

The goal of cost estimation was to quantify the cost of treatment of each disease, not necessarily over a fixed time period, but rather according to clinical presentation, ie, per case. No national nomenclature of treatment costs is available in Algeria, so several public (n=3) and private (n=7) institutions were contacted by phone to obtain local cost information in DZD. The collected costs for each diagnostic test and treatment were aggregated to estimate the total cost of diagnosis, treatment, and management of recurrence of cervical cancer, CIN, and genital warts. Costs for hospitalization and outpatient visits were estimated for cervical cancer and CIN only.

The specific questions used to calculate the costs are shown in **Supplementary Table S1.** As the numbers of patients at each institution were not available, cost analyses were performed for a single hypothetical case at each institution and averaged across all participating institutions. For questionnaire items with 4 possible answers, the following frequencies were assumed: never, zero patients; sometimes, 25% of patients; regularly, 50% of patients; always, 100% of patients. Therefore, the cost per patient of each diagnostic test and treatment was adjusted by multiplying the individual cost by 0, 0.25, 0.5, or 1 depending on the answer to the item by the physician. Costs of diagnosis, treatment, and recurrence are reported per patient, and costs for hospitalizations and outpatient visits are reported per institution per year.

## RESULTS

### Participant Characteristics

All 15 experts agreed to participate in the study. The median (range) age of panel participants was 54 (40-74) years. Eleven panelists were male (73%), and 4 were female (27%). Specialties included gynecology, oncology, and dermatology (n=5 each, 33%). The median (range) time in medical practice was 30 (12-48) years.

### Practice Characteristics

Among gynecologists and oncologists, it was estimated that 11% of their monthly caseload involved patients with CIN/cervical cancer **(Table 1).** Panelists estimated that 18% of the CIN patients were at stage 1, 28% were at stage 2, and 23% were at stage 3. The median duration of care for a single patient with CIN/cervical cancer was 10 years. Among CIN patients, 10% were under age 30, and among cervical cancer patients, 18% were under age 40. Panelists estimated the incidence of cervical cancer in Algeria to be 2800 cases per year.

**Table 1.** Practice Characteristics^a^

**Characteristics**	
Cervical cancer, CIN	n=10
Percentage of total patients per month	11% (10-20)
CIN stage	
1	18% (15-50)
2	28% (15-40)
3	23% (10-30)
Duration of follow-up per case, years	10 (5-24)
Age distribution	
CIN patients <30 years	10% (5-20)
Cervical cancer patients <40 years	18% (10-25)
Physician-estimated incidence of cervical cancer, cases per year	2,800 (1,500-4,000)
Genital warts	n=5
Percentage of total patients per month	4% (2-5)
Duration of medical practice treating patients with genital warts, years	25 (17-38)
Distribution of patients	
Male	65% (60-66)
Female	35% (34-40)
Aged <20 years	13% (10-20)
First presentation	70% (50-90)

Abbreviation: CIN, cervical intraepithelial neoplasia.

^a^ Values are presented as median (interquartile range).

Among dermatologists, it was estimated that 4% of their monthly caseload involved patients with genital warts **(Table 1).** The median time in clinical practice treating patients with genital warts was 25 years. Among patients with genital warts, an estimated 65% were men and 35% were women, and 13% were under age 20. A median of 70% of patients were presenting with genital warts for the first time. Panelists estimated the prevalence of genital warts at their institution to range from 2% to 8% in men and 2% to 3% in women.

### Diagnostic Tests

**Figure 1.**
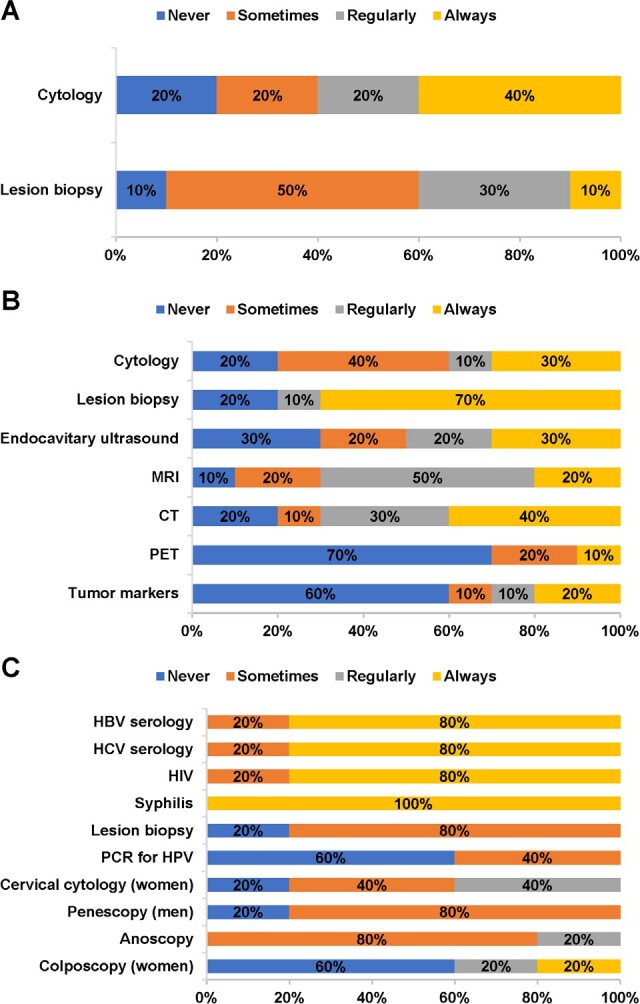
Diagnostic Tests for (A) CIN, (B) Cervical Cancer, and (C) Genital Warts Abbreviations: CIN, cervical intraepithelial neoplasia; CT, computed tomography; HBV, hepatitis B virus; HCV, hepatitis C virus; HIV, human immunodeficiency virus; HPV, human papillomavirus; MRI, magnetic resonance imaging; PCR, polymerase chain reaction; PET, positron emission tomography.

Among gynecologists and oncologists, 60% reported that cytology tests were performed regularly or always for the diagnosis of CIN, and 40% said the same about lesion biopsies **(Figure 1A).** For the diagnosis of cervical cancer, lesion biopsies, magnetic resonance imaging (MRI), and computed tomography (CT) scans were the most common diagnostic tests, according to the percentage of panelists ranking them as being performed regularly or always **(Figure 1B).** Forty percent of panelists reported that systematic screening for CIN/cervical cancer was performed regularly or always (not shown). Systematic screening typically occurred 2-3 times per year in women of median age 25 (IQR, 25-35).

Among dermatologists, diagnostic tests reported to be performed regularly or always in patients with genital warts included assays for hepatitis B, hepatitis C, HIV, and syphilis; anoscopy; and, in women, cervical cytology tests and colposcopy **(Figure 1C).** Most panelists reported that revision (ie, a control visit) was conducted (80%) and laboratory tests were requested (60%) regularly or always for the sexual partners of patients with genital warts (not shown).

### Availability of Treatment Guidelines and Resources

Most gynecologists and oncologists (60%) reported the availability of treatment guidelines for CIN/cervical cancer at their institution, and 80% reported the availability of all resources needed to treat CIN/cervical cancer according to the guidelines. In contrast, most dermatologists (80%) reported that treatment guidelines for genital warts were not available at their institution, with 3 of 4 reporting that at least some resources for treating genital warts were lacking.

### Outpatient Visits and Hospitalizations

Gynecologists and oncologists estimated an institution-wide total of 20 outpatient visits (to oncologists or similar health care providers) per year for CIN and 50 outpatient visits per year for cervical cancer **(Table 2).** The number of hospitalizations per year was estimated at 15 for CIN and 11 for cervical cancer. The estimated length of hospitalization was 1 day for CIN and 7 days for cervical cancer. Panelists reported an average of 4 follow-up visits for CIN/cervical cancer per patient per year.

**Table 2.** Outpatient Visits and Hospitalizations for Cervical Cancer or CIN^a^

**Health Care Resource Use**	**(n=10)**
Outpatient visits to oncologists per year	
CIN	20 (0-20)
Cervical cancer	50 (15-150)
Hospitalizations per year	
CIN	15 (0-50)
Cervical cancer	11 (10-80)
Length of hospital stay, days	
CIN	1 (0-3)
Cervical cancer	7 (4-10)

Abbreviation: CIN, cervical intraepithelial neoplasia.

^a^ Values are presented as median (interquartile range).

Among dermatologists, the majority (60%) reported that patients with genital warts were never admitted to the hospital for treatment, although 2 panelists reported that hospitalization occurred sometimes (n=1) or always (n=1). Panelists estimated that patients with genital warts had an average of 12 follow-up visits per year.

### Treatments and for Initial and Recurring Cases

**Figure 2.**
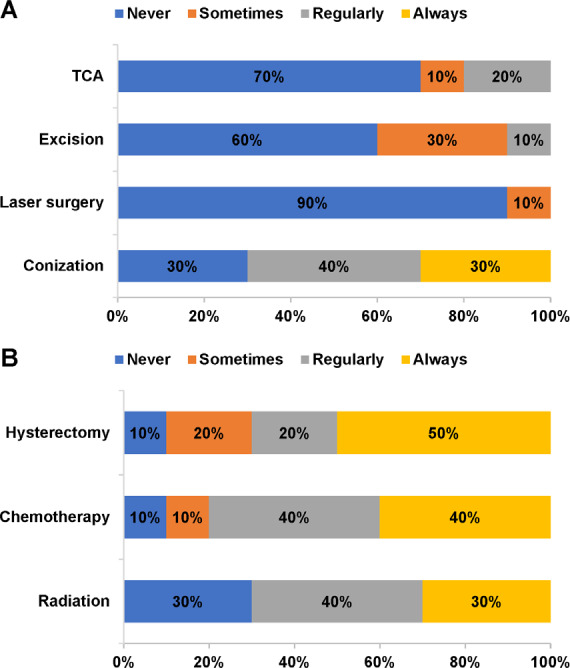
Treatments for (A) CIN and (B) Cervical Cancer Abbreviations: CIN, cervical intraepithelial neoplasia; TCA, trichloroacetic acid.

For CIN, 70% of gynecologists and oncologists reported that conization was regularly or always used as a treatment **(Figure 2A).** The majority of panelists reported that large loop excision of the transformation zone (60%), trichloroacetic acid (70%), and laser surgery (90%) were never used as treatments for CIN. Treatments used regularly or always for cervical cancer included chemotherapy (80%), hysterectomy (70%), and radiation (70%) **(Figure 2B).** According to 80% of panelists, complications of treatment for CIN/cervical cancer occurred “sometimes.” Complications occurring sometimes or regularly included bleeding (90%) and infection (100%). Recurrence of disease after treatment for CIN/cervical cancer occurred sometimes (80%) or regularly (10%). For recurrence of CIN, conization (40%) and hysterectomy (40%) were the treatments used regularly or always **(Supplementary Figure S1A).** For recurrence of cervical cancer, hysterectomy, chemotherapy, and radiation became less frequent than during initial treatment but were still used by the majority of panelists at least sometimes **(Supplementary Figure S1B).**

**Figure 3.**
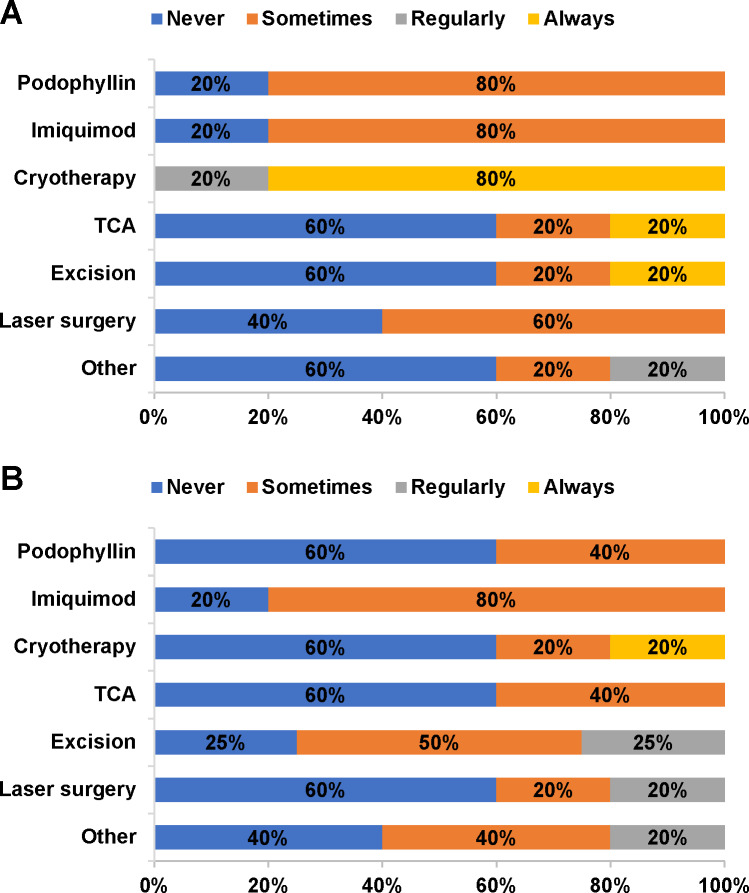
(A) First-line and (B) Second-line Treatments for Genital Warts^a^ Abbreviation: TCA, trichloroacetic acid. ^a^ n=4 for excision as second-line treatment.

For genital warts, 100% of dermatologists reported cryotherapy to be the first-line treatment of choice (regularly or always; **Figure 3A).** Cryotherapy remained in routine use as a second-line therapy (20% always, 20% sometimes), but excision and laser surgery became second-line options for regular use by 25% and 20% of panelists, respectively **(Figure 3B).** According to 60% of panelists, complications occurred sometimes or regularly in patients treated for genital warts. Complications included bleeding (40% regularly), infection (60% sometimes), and deformity (40% sometimes). Recurrence after treatment for genital warts was reported to occur sometimes (40%) or regularly (40%). All panelists reported using cryotherapy (40% regularly, 60% always) for recurring cases of genital warts **(Supplementary Figure S1C).**

### Referrals

According to gynecologists/oncologists, patients with CIN/cervical cancer are referred to other specialties sometimes (70%) or always (20%). Specialties to which these panelists refer patients with CIN/cervical cancer regularly or always include surgeons (10%), gynecologists (50%, all from oncologists), and psychologists (40%). All dermatologists sometimes (80%) or regularly (20%) referred patients with genital warts to other specialists, generally to other dermatologists or to gynecologists.

### Costs

The estimated costs for diagnosis, treatment, and recurrence of CIN/cervical cancer and genital warts are shown in Table 3. Overall, costs for cervical cancer were higher than for the other diseases with median (IQR) costs of 53 750 DZD (31 500-74 750) for cervical cancer, 6750 DZD (4750-11 500) for CIN, and 16 075 DZD (14 350-16 325) for genital warts. Initial treatment costs were higher than recurrence costs for cervical cancer (650 000 DZD versus 431 250 DZD), whereas recurrence incurred higher costs than initial treatment for CIN (77 750 DZD vs 19 750) and genital warts (9250 DZD vs 7500-8000 DZD). Hospitalization contributed the bulk of annual costs for cervical cancer (975 000 DZD), whereas outpatient visits represented a larger proportion of the annual costs of CIN (60 000 DZD).

**Table 3.** Costs Associated With Cervical Cancer, CIN, and Genital Warts in Algeria^a^

	**Cervical Cancer**	**CIN**	**Genital Warts**
Diagnosis, per patient	53 750 (31 500-74 750)	6750 (4750-11 500)	16 075 (14 350-16 325)
Initial treatment, per patient	650 000 (350 000-1 050 000)	19 750 (0.00-35 000)	First line: 8000 (7750-11 125) Second line: 7000 (6000-8375)
Treatment for recurrence, per patient	431 250 (312 500-550 000)	77 750 (8750-117 500)	9250 (8750-11 125)
Outpatient visits, per year	150 000 (45 000-450 000)	60 000 (0-60 000)	—
Hospitalizations, per year	975 000 (525 000-1 500 000)	50 000 (0-150 000)	—

Abbreviation: CIN, cervical intraepithelial neoplasia.

^a^ Costs are reported as median (interquartile range) in DZD.

## DISCUSSION

This study gives a first estimation of the diagnosis and treatment patterns, HCRU, and costs associated with cervical cancer, CIN, and genital warts in Algeria. Diagnosis and treatment patterns and HCRU estimates were obtained from a panel of experts practicing in Algeria, and total costs were calculated by synthesizing panelist responses with public and private data sources on individual cost line items.

The diagnosis and treatment patterns reported by the panelists in this study were generally in line with international recommendations. The European Society for Medical Oncology’s guidelines on the diagnosis, treatment, and follow-up of cervical cancer state that colposcopy, biopsy, or excisional procedures are appropriate responses to an abnormal cytology result or a positive HPV test.^12^ Upon histological confirmation of cancer, first-line treatment options are conization, simple hysterectomy, or radical hysterectomy.^12^ MRI and CT are among the techniques used to visualize the tumor. Radiation and chemotherapy are available as adjuvants to surgery in specific cases.^12^ In the current study, a majority of the panelists reported using biopsy, MRI, and CT regularly or always for the diagnosis of cervical cancer, in accordance with the guidelines. Similarly, hysterectomy, chemotherapy, and radiation were all used regularly or always by a majority of panelists to treat cervical cancer.

The 2019 International Union Against Sexually Transmitted Infections (IUSTI)-Europe guideline for the management of anogenital warts recommends visual inspection as the best method for diagnosis, stating that biopsy and HPV testing are not necessary.^13^ The practices of the panelists in the current study were in line with these recommendations; no physicians used biopsy and HPV testing more than “sometimes,” and diagnostic tests generally focused on a broader assessment of sexually transmitted and other infections. For treatment, the IUSTI-Europe guideline recommends topical agents and surgical removal.^13^ Cryotherapy is another recommended treatment, with clearance rates equal to or better than chemical agents.^13^ In the current study, cryotherapy was the most frequently used treatment for genital warts.

In estimates of HCRU for genital warts in Algeria, most panelists reported that patients with genital warts were never admitted to the hospital and that each case of genital warts required an average of 12 follow-up visits per year. A systematic review of studies published through 2010 found that physician visits per genital warts episode ranged from 2.8 to 4.7.^14^ Several publications thereafter confirm this range.^15–17^

The total estimated cost per patient with genital warts in Algeria (diagnosis, first- and second-line treatment, and recurrence combined) was 40 825 DZD, which is equivalent to US $308 (using an online currency converter on April 13, 2021). The systematic review mentioned above reported a range of costs per episode of genital warts in different countries, from $168 to $1196 (in 2009 US dollars).

The panelists in this study provided estimates of the incidence of cervical cancer and the prevalence of genital warts in Algeria. Other sources of data on cervical cancer in Algeria include the HPV Information Centre^11^ and the GLOBOCAN series of analyses.^9,18,19^ Based on the GLOBOCAN 2020 fact sheet, a total of 1663 new cases of cervical cancer were observed in 2020, with a 5-year prevalence of 4499 across all age groups.^20^ The incidence estimate given by the panelists in this study (2800 cases per year) falls within these two values, suggesting that the participating physicians had an accurate view of the annual caseload. According to the HPV Information Centre, the incidence of cervical cancer in Algeria peaks in women aged 50-60,^11^ which is consistent with the panelists’ estimates of age in the current study (10% of CIN patients under age 30, 18% of cervical cancer patients under age 40). To our knowledge, there are no extant data on genital warts in Algeria, but the estimate of 2% to 8% prevalence in men and 2% to 3% prevalence in women is consistent with reported values from other African countries.^21–23^

According to the HPV Information Centre, Algeria has a national cervical cancer screening program.^11^ In this program, screening is conducted every 3 years starting at age 25 or 30, and cytology is the primary diagnostic test used. Responses from the panelists in the current study suggest that this screening program is not being implemented to its full extent, since just 40% reported that systematic screening for CIN/cervical cancer was performed regularly or always. However, this is an improvement over data from 2001-2007 showing that screening coverage was at just 2% of the target population.^24^ In addition, the median age at screening reported by the panelists in the current study (25 years) matches the program’s specifications, and 60% of panelists agreed that cytology tests were performed regularly or always for the diagnosis of CIN. Algeria does not have a formal HPV vaccination program. However, like most North African countries, it has vaccination programs for communicable diseases including tuberculosis, diphtheria, pertussis, tetanus, polio and measles.^25^ Algeria also vaccinates against influenza (in the elderly) and hepatitis B. Thus, there is potential for HPV vaccination to be implemented along with current vaccination strategies. Barriers to HPV vaccination include lack of awareness or acceptance of the vaccine,^26^ and limited financial and infrastructural resources.^25^

Considering the scarcity of data from Algeria on the management of HPV-related diseases, the current study provides valuable new and novel information on the prevalence of genital warts and the management of cervical cancer, CIN, and genital warts. The panelists’ answers offer insight into the current diagnosis and treatment patterns for cervical cancer, CIN, and genital warts in Algeria, as well as estimates of the resource use associated with each condition.

Despite these strengths, this study has several limitations. First and foremost, the results are based on answers to a questionnaire that, due to lack of other data, could not be compared or verified against other sources such as electronic medical records or health insurance databases. However, we note that previous studies of the HCRU and costs associated with warts in other countries have used expert opinion as a data source.^16,17^ In addition, the results reported here are from a panel of 15 oncologists, gynecologists, and dermatologists so the results may not be representative of trends observed in the entire country. Second, this study did not calculate the actual economic burden of HPV-related diseases in Algeria but rather estimated the costs based on the answers to the questionnaire plus local cost data. It is highly likely that the costs were underestimated, as costs related to laboratory tests and nursing were not included. The estimated costs reported here likely represent the minimum of anticipated costs. Third, the study design is prone to bias via the selection of experts who could underestimate or overestimate the quantity of cervical cancer, CIN, and genital warts in Algeria, or report management practices that are influenced by their field of specialty. To minimize this bias, we selected experts in equal numbers from each specialty and emphasized the responses that reached consensus. Fourth, HCRU and costs were not stratified by severity of disease.

In conclusion, this study investigated the management of patients with cervical cancer, CIN, or genital warts in Algeria and provided the first estimates of diagnosis and treatment patterns, HCRU, and costs associated with these conditions. These resource use and cost estimates highlight the need to develop prevention strategies for HPV-related pathologies.

## Supplementary Material

Appendix: Questionnaire

Online Supplemental Material

## References

[ref-105043] Bruni L., Albero G., Serrano B.. (2019). Human Papillomavirus and Related Diseases in the World.

[ref-105044] Insinga Ralph P, Dasbach Erik J, Elbasha Elamin H (2009). Epidemiologic natural history and clinical management of Human Papillomavirus (HPV) Disease: a critical and systematic review of the literature in the development of an HPV dynamic transmission model. BMC Infectious Diseases.

[ref-105045] Lee Lai-Yang, Garland Suzanne M. (2017). Human papillomavirus vaccination: the population impact. F1000Research.

[ref-105046] Peirson Leslea, Fitzpatrick-Lewis Donna, Ciliska Donna, Warren Rachel (2013). Screening for cervical cancer: a systematic review and meta-analysis. Systematic Reviews.

[ref-105047] Jansen Erik E.L., Zielonke Nadine, Gini Andrea, Anttila Ahti, Segnan Nereo, Vokó Zoltán, Ivanuš Urška, McKee Martin, de Koning Harry J., de Kok Inge M.C.M., Veerus Piret, Anttila Ahti, Heinävaara Sirpa, Sarkeala Tytti, Csanádi Marcell, Pitter Janos, Széles György, Vokó Zoltán, Minozzi Silvia, Segnan Nereo, Senore Carlo, van Ballegooijen Marjolein, Driesprong - de Kok Inge, Gini Andrea, Heijnsdijk Eveline, Jansen Erik, de Koning Harry, Lansdorp – Vogelaar Iris, van Ravesteyn Nicolien, Zielonke Nadine, Ivanus Urska, Jarm Katja, Mlakar Dominika Novak, Primic-Žakelj Maja, McKee Martin, Priaulx Jennifer (2020). Effect of organised cervical cancer screening on cervical cancer mortality in Europe: a systematic review. European Journal of Cancer.

[ref-105048] Bray Freddie, Ferlay Jacques, Soerjomataram Isabelle, Siegel Rebecca L., Torre Lindsey A., Jemal Ahmedin (2018). Global cancer statistics 2018: GLOBOCAN estimates of incidence and mortality worldwide for 36 cancers in 185 countries. CA: A Cancer Journal for Clinicians.

[ref-105049] Vaccarella Salvatore, Bruni Laia, Seoud Muhieddine (2013). Burden of human papillomavirus infections and related diseases in the extended Middle East and North Africa region. Vaccine.

[ref-105050] Bruni Laia, Diaz Mireia, Castellsagué Xavier, Ferrer Elena, Bosch F. Xavier, de Sanjosé Silvia (2010). Cervical human papillomavirus prevalence in 5 continents: meta-analysis of 1 million women with normal cytological findings. The Journal of Infectious Diseases.

[ref-105051] Ferlay Jacques, Shin Hai-Rim, Bray Freddie, Forman David, Mathers Colin, Parkin Donald Maxwell (2010). Estimates of worldwide burden of cancer in 2008: GLOBOCAN 2008. International Journal of Cancer.

[ref-105052] Kim J.J., Sharma M., O’Shea M.. (2013). Model-based impact and cost-effectiveness of cervical cancer prevention in the Extended Middle East and North Africa (EMENA). Vaccine.

[ref-105053] Bruni L., Albero G., Serrano B.. (2019). Human Papillomavirus and Related Diseases in Algeria.

[ref-105054] Marth C., Landoni F., Mahner S., McCormack M., Gonzalez-Martin A., Colombo N. (2017). Cervical cancer: ESMO Clinical Practice Guidelines for diagnosis, treatment and follow-up. Annals of Oncology.

[ref-105055] Gilson R., Nugent D., Werner R.N., Ballesteros J., Ross J. (2020). 2019 IUSTI-Europe guideline for the management of anogenital warts. Journal of the European Academy of Dermatology and Venereology.

[ref-105056] Raymakers Adam J.N., Sadatsafavi Mohsen, Marra Fawziah, Marra Carlo A. (2012). Economic and humanistic burden of external genital warts. Pharmacoeconomics.

[ref-105057] Salo Heini, Leino Tuija, Kilpi Terhi, Auranen Kari, Tiihonen Petri, Lehtinen Matti, Vänskä Simopekka, Linna Miika, Nieminen Pekka (2013). The burden and costs of prevention and management of genital disease caused by HPV in women: a population-based registry study in Finland. International Journal of Cancer.

[ref-105058] Buenconsejo Lani, Kothari-Talwar Smita, Yee Karen, Kulkarni Amit, Lara Nuria, Roset Montserrat, Giuliano Anna R., Garland Suzanne (2019). Estimating the burden of illness related to genital warts in the Philippines: a nationally representative cross-sectional study. Infectious Agents and Cancer.

[ref-105059] Lee Taek Sang, Kothari-Talwar Smita, Singhal Puneet K, Yee Karen, Kulkarni Amit, Lara Nuria, Roset Montserrat, Giuliano Anna R, Garland Suzanne M, Ju Woong (2017). A cross-sectional study estimating the burden of illness related to genital warts in South Korea. BMJ Open.

[ref-105060] Ferlay J., Colombet M., Soerjomataram I., Mathers C., Parkin D.M., Piñeros M., Znaor A., Bray F. (2019). Estimating the global cancer incidence and mortality in 2018: GLOBOCAN sources and methods. International Journal of Cancer.

[ref-105061] Ferlay Jacques, Soerjomataram Isabelle, Dikshit Rajesh, Eser Sultan, Mathers Colin, Rebelo Marise, Parkin Donald Maxwell, Forman David, Bray Freddie (2015). Cancer incidence and mortality worldwide: sources, methods and major patterns in GLOBOCAN 2012. International Journal of Cancer.

[ref-105062] WHO International Agency for Research on Cancer (2020). Algeria fact sheet.

[ref-105063] Dareng Eileen O., Adebamowo Sally N., Famooto Ayotunde, Olawande Oluwatoyosi, Odutola Michael K., Olaniyan Yinka, Offiong Richard A., Pharoah Paul P., Adebamowo Clement A. (2019). Prevalence and incidence of genital warts and cervical Human Papillomavirus infections in Nigerian women. BMC Infectious Diseases.

[ref-105064] De Vuyst Hugo, Alemany Laia, Lacey Charles, Chibwesha Carla J., Sahasrabuddhe Vikrant, Banura Cecily, Denny Lynette, Parham Groesbeck P. (2013). The burden of human papillomavirus infections and related diseases in sub- Saharan Africa. Vaccine.

[ref-105065] Low Andrea J, Clayton Tim, Konate Issouf, Nagot Nicolas, Ouedraogo Abdoulaye, Huet Charlotte, Didelot-Rousseau Marie-Noelle, Segondy Michel, Van de Perre Philippe, Mayaud Philippe (2011). Genital warts and infection with human immunodeficiency virus in high-risk women in Burkina Faso: a longitudinal study. BMC Infectious Diseases.

[ref-105066] Sancho-Garnier Hélène, Khazraji Youssef Chami, Cherif Moktar Hamdi, Mahnane Abbes, Hsairi Mohamed, Shalakamy Amr El, Osgul Nejat, Tuncer Murat, Jumaan Aisha O., Seoud Muhieddine (2013). Overview of cervical cancer screening practices in the extended Middle East and North Africa countries. Vaccine.

[ref-105067] Jumaan Aisha O., Ghanem Soha, Taher Jalaa, Braikat Mhammed, Awaidy Salah Al, Dbaibo Ghassan S. (2013). Prospects and challenges in the introduction of human papillomavirus vaccines in the Extended Middle East and North Africa region. Vaccine.

[ref-105068] Gamaoun Rihab (2018). Knowledge, awareness and acceptability of anti-HPV vaccine in the Arab states of the Middle East and North Africa Region: a systematic review. Eastern Mediterranean Health Journal.

