# Supplementary material for: Costs and Resource Use Among Patients with Cervical Cancer, Cervical Intraepithelial Neoplasia, and Genital Warts in Algeria
Source: J Health Econ Outcomes Res. 2022 Feb 7;9(1):31–8. doi: 10.36469/jheor.2022.31049 (PMC8820946; doi:10.36469/jheor.2022.31049)
Supplement: Online Supplemental Material [file jheor_2022_9_1_31049_81466.pdf]

Journal of Health Economics and Outcomes Research

Online Supplementary Material

Costs and Resource Use Among Patients With Cervical Cancer, Cervical Intraepithelial Neoplasia, and Genital Warts in Algeria. *JHEOR*. 2021;9(1):11-18. doi:10.36469/jheor.2022.31049.

Figure S1. Treatment for Recurrent Cases of (A) Cervical Intraepithelial Neoplasia, (B) Cervical Cancer, and (C) Genital Warts

Table S1. Source of Cost Data

Appendix. Questionnaire

This supplementary material has been provided by the authors to give readers additional information about their work.

This is an open-access article distributed under the terms of the Creative Commons Attribution 4.0 International License (CCBY-4.0). View this license's legal deed at http://creativecommons.org/licenses/by/4.0 and legal code at http://creativecommons.org/licenses/by/4.0/legalcode for more information.

Figure S1. Treatment for Recurrent Cases of (A) Cervical Intraepithelial Neoplasia, (B) Cervical Cancer, and (C) Genital Warts

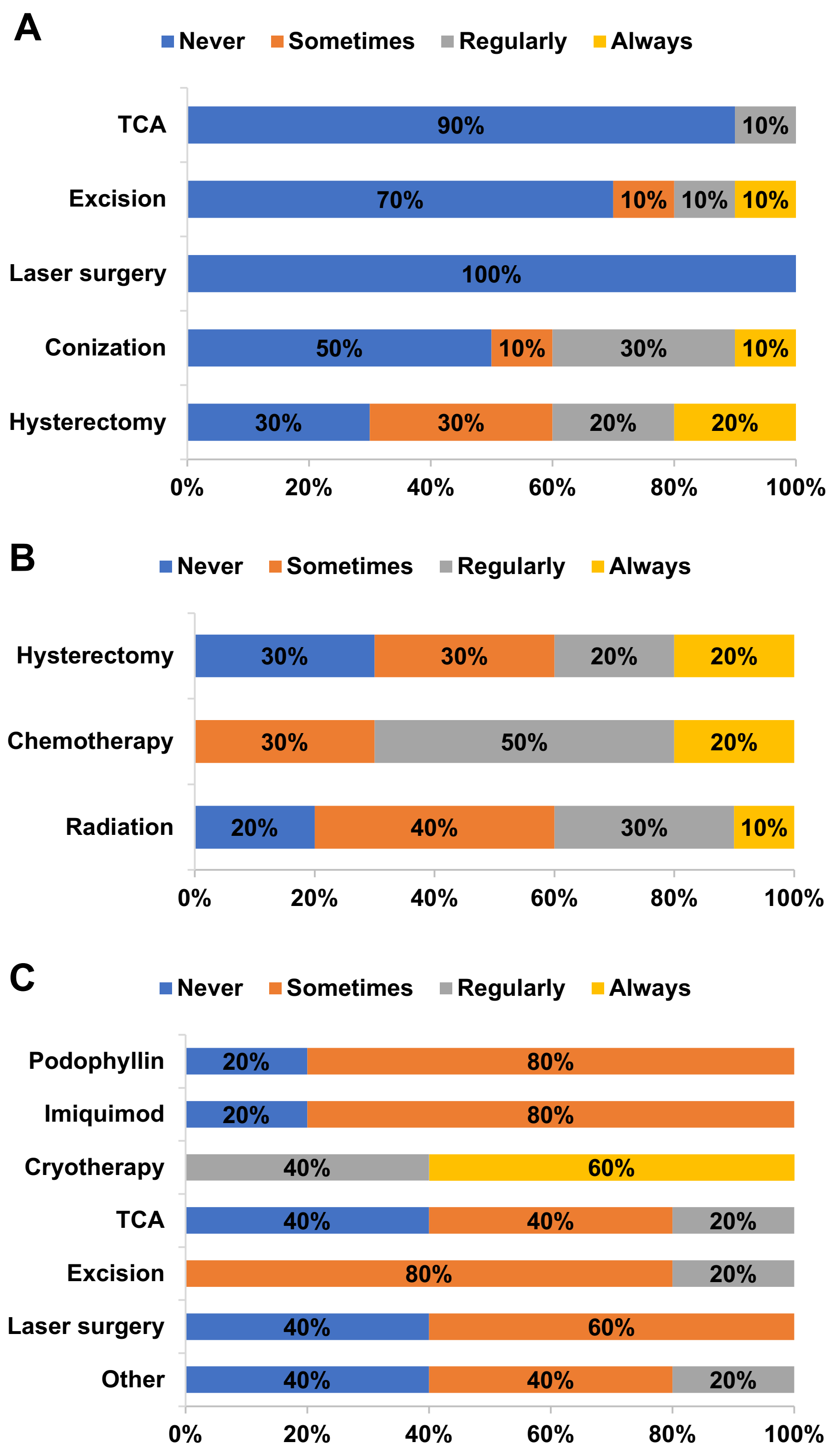

CIN, cervical intraepithelial neoplasia; TCA, trichloroacetic acid.

Table S1. Source of Cost Data

Cost Type	Questionnaire Item	Frequency Data
Cervical cancer		
Cost of diagnosing patient with cervical cancer	Section II, Question 8B	Figure 1B
Cost of treatment of patient with cervical cancer	Section III, Question 6B	Figure 2B
Cost of treatment of patient with recurrence from cervical cancer	Section III, Question 13B	Figure S1B
Annual cost of outpatient visits to oncologist and similar	Section III, Question 3	—
Annual cost of hospitalization for cervical cancer	Section III, Question 5	—
CIN		
Cost of diagnosing patient	Section II, Question 8A	Figure 1A
Cost of treatment of patient	Section III, Question 6A	Figure 2A
Cost of treatment of patient with recurrence from CIN	Section III, Question 13A	Figure S1A
Annual cost of outpatient visits to oncologist and similar	Section III, Question 3	-—
Annual cost of hospitalization for cervical cancer	Section III, Question 5	-—
Genital warts		
Cost of diagnosing patient	Section II, Questions 11-15	Figure 1C
Cost of first-line treatment of patient	Section III, Question 4A	Figure 3A
Cost of second-line treatment of patient	Section III, Question 4B	Figure 3B
Cost of treatment of patient with recurrence from genital warts	Section III, Question 13	Figure S1C

CIN, cervical intraepithelial neoplasia.

Appendix. Questionnaire

Section	Question	Cervical Cancer, CIN	Genital Warts
II	1	In average, what is the percentage of patients with cervical cancer, CIN1/2/3 do you offer care per month?	In average, to how many patients with genital warts do you offer care per month? (% of total)
	2	What is the total length of care provided to patients with cervical cancer or CIN1/2/3 (total follow-up length)?	Time offering care to patients with genital warts (years):
	3	From your patients with CIN lesions, what is the percentage for each stage?	From your patients with genital warts, what percentage are (a) male, (b) female?
	4	From your patients with CIN1/2/3, what percentage is younger than 30 years of age?	From your patients with genital warts, what percentage is younger than 20 years of age?
	5	From your patients with cervical cancer, what percentage is younger than 40 years of age?	From your patients with anogenital warts, what percentage presents the condition for the first time?
	6	In France, the incidence of cervical cancer is 2840 cases/year What do you consider to be the incidence of cervical cancer in Algeria?	From your patients with anogenital warts for the first time, what percentage are (a) male, (b) female?
	7	At your institution, what department(s) offers care to patients with (a) CIN, (b) cervical cancer?	From your patients with anogenital warts for the first time, what percentage is younger than 20 years of age?
	8	At your institution what tests are ordered /performed for the diagnosis and costs? (never, sometimes, regularly, always)	In the USA, the incidence of genital warts in men ranges between 2% and 11%, while in women, ranges between 1% and 3%. What do you consider to be the prevalence of genital warts in your institution?
		(A) CIN: cytology, lesion biopsy	
		(B) Cervical cancer: cytology, lesion biopsy, endocavitary ultrasound, MRI, CT, PET, tumor markers	
	9	At your institution is CIN1,2,3 and cervical cancer screening systematically practiced? (never, sometimes, regularly, always)	At your institution, what department(s) offers care to patients with genital warts?
	10	If yes, at what age (years) it will be practiced and at what rhythm (every # years)?	In addition to the clinical review, does your institution conduct diagnosis tests to patients with anogenital warts? (never, sometimes, regularly, always)
	11	--	What tests are ordered at your institution: HBV serology, HCV serology, ELISA for HIV, VDRL, lesion biopsy, PCR for HPV, cervical cytology (women only), penescopy (men only), anoscopy, other? (never, sometimes, regularly, always)
	12	--	In women with anogenital warts or condyloma acuminata, is a colposcopy conducted at your institution? (never, sometimes, regularly, always)
	13	--	At your institution, when is a biopsy requested from your patients with anogenital warts? (never, sometimes, regularly, always)

	14	--	At your institution, is a revision conducted in sexual partners of patients with anogenital warts? (never, sometimes, regularly, always)
	15	--	At your institution, are laboratory tests requested from sexual partners of patients with anogenital warts? (never, sometimes, regularly, always)
III	1	At your institution, are there available clinical practice guidelines for the treatment cervical cancer and CIN1/2/3? (yes, no)	At your institution, are there available clinical practice guidelines for the treatment of anogenital warts? (yes, no)
	2	Do you have available all resources for the treatment of cervical cancer and CIN1/2/3 as per the guidelines?	Do you have available all resources for the treatment of genital warts as per the guidelines?
	3	At your institution, what is average number of outpatient visits to oncologist and similar by year for (a) CIN and (b) cervical cancer?	At your institution, are patients with anogenital warts prescribed any specific type of treatment? (never, sometimes, regularly, always)
	4	At your institution, what is average number of hospitalizations by year for (a) CIN and (b) cervical cancer?	(A) At your institution, what therapies are considered as the first line of treatment for anogenital warts: podophyllin, imiquimod, cryotherapy, TCA, excision, laser surgery? (never, sometimes, regularly, always)
			(B) At your institution, what therapies are considered as the second line of treatment for anogenital warts: podophyllin, imiquimod, cryotherapy, TCA, excision, laser surgery? (never, sometimes, regularly, always)
